# Maize-soybean intercropping facilitates chemical and microbial transformations of phosphorus fractions in a calcareous soil

**DOI:** 10.3389/fmicb.2022.1028969

**Published:** 2022-11-16

**Authors:** Jin Liu, Yang Li, Chaoqun Han, Dongling Yang, Jianjun Yang, Barbara J. Cade-Menun, Yuanquan Chen, Peng Sui

**Affiliations:** ^1^College of Agronomy and Biotechnology, China Agricultural University, Beijing, China; ^2^Institute of Environment and Sustainable Development in Agriculture, Chinese Academy of Agricultural Sciences, Beijing, China; ^3^Agriculture and Agri-Food Canada, Swift Current RDC, Swift Current, SK, Canada

**Keywords:** legacy P, mobilization, rhizosphere effect, phosphatase, P-solubilizing bacteria

## Abstract

Intercropping often substantially increases phosphorus (P) availability to plants compared with monocropping, which could be an effective strategy for soil legacy P recovery and agricultural production. However, the biogeochemical interactions among plants, microbes, and soil that mobilize P remain largely unknown in intercropping systems. Pot experiments with maize-soybean intercropping in a calcareous soil were conducted to investigate the potential chemical and biological transformation mechanisms of inorganic P (P_i_) and organic P (P_o_) using sequential extraction and Illumina MiSeq sequencing. Compared to monocropping of each crop, maize-soybean intercropping significantly enhanced total P uptake of the two crops by mobilizing Ca_2_-P_i_ [extracted by bicarbonate (NaHCO_3_)], Al-P_i_/P_o_ [extracted by ammonium fluoride (NH_4_F)] and Fe-P_i_ [extracted by sodium hydroxide and sodium carbonate (NaOH-Na_2_CO_3_)] fractions. Furthermore, there were significant increases in the organic carbon content and alkaline phosphomonoesterase (ALP) and phosphodiesterase (PDE) activities as well as the abundances of *Microvirga*, *Lysobacter*, *Microlunatus* and *Sphingomonas* under maize-soybean intercropping relative to monocropping. In contrast, compared to monocroppping, no significant change in the soil pH was observed under maize-soybean intercropping. Therefore, the enhanced P uptake of the maize-soybean intercropping probably resulted from a synergistic effect of rhizosphere organic carbon deposit, increased activities of ALP and PDE, together with the bacteria (*Microvirga*, *Lysobacter*, *Microlunatus* and *Sphingomonas*) which showed correlation with soil P forms, while the generally recognized rhizosphere acidification was excluded in this investigated calcareous soil. Moreover, the selected bacterial genera exhibited a closer network in the rhizosphere of soybean compared to maize, suggesting enhanced interactions among bacteria in the soybean rhizosphere. These results provide theoretical bases for the recovery of soil legacy P by maize-soybean intercropping.

## Introduction

Phosphorus (P) limits crop yield on more than 30% of the world’s arable land (Vance et al., 2003). Widespread use of chemical phosphate fertilizer has substantially depleted phosphate rock resources, and also has led to environmental pollution due to loss of fertilizer P from agricultural land to water bodies (Sattari et al., 2012). Traditional fertilization practices generally result in the accumulation of high P concentrations in soil. It is estimated that 15% of applied P is absorbed by plants, but 60% remains in the topsoil (up to 15 cm depth) in forms that are not readily available to plants (Wagar et al., 1986). There is a growing recognition that the recovery of this residual or legacy soil P could reduce the reliance on chemical fertilizers and their negative impacts on environment (Sattari et al., 2012; Liu et al., 2015; Withers et al., 2019).

Crop intercropping, growing more than one crop simultaneously, has been shown to increase crop yields and nutrient acquisition (Li et al., 2007; Tian et al., 2020). The intercropping of cereals with legumes, widely recognized to enhance soil nitrogen (N) fertility, could facilitate the recovery of soil legacy P (Li et al., 2007). For example, previous studies indicated intercropping with legumes mobilized more soil P compared to single-cropped maize and wheat (Liao et al., 2020; Messaoudi et al., 2020). Since the bioavailability of soil P compounds depends on their chemical forms (Liu et al., 2013), the recovery of soil P by intercropping was mainly through root-induced transformation of specific soil P pools or fractions. Soil P speciation greatly depends on soil type and land use (Negassa and Leinweber, 2009; Liu et al., 2018), and previous studies have shown that different types of crops access different soil P pools. For example, maize could readily deplete the NaOH-extractable inorganic P (NaOH-P_i_) fraction determined by the Hedley fractionation method (Cabeza et al., 2017), while in a monocropping study, soybean enriched the NaHCO_3_-extracted organic P (P_o_) fraction, but had less depletion of the NaOH-P_i_ fraction compared with maize (Rubio et al., 2012). In intercropping studies of maize and faba bean, more residual-P fraction was depleted under intercropping compared to monocropping after a 4-year study withholding nitrogen (N) and P fertilization (Liao et al., 2020). However, the transformation of soil P fractions under maize-soybean intercropping remains largely unknown.

Multiple strategies to mobilize soil P through the transformation of different legacy P fractions in agricultural soils exist in cereal-legume intercropping systems, including root-induced pH changes, root exudates, and enhanced phosphatase and microbial activities (Hinsinger, 2001; Roohi et al., 2020). Tang et al. (2016) found microbial biomass P significantly increased in the rhizosphere of faba bean when intercropped with durum wheat, which highlighted the potential involvement of microbes in rhizospheric P cycling. Bargaz et al. (2017) reported that the soil microbial community shaped by the rhizosphere effect of wheat under wheat-soybean intercropping could promote P uptake of wheat. Recently, Roohi et al. (2020) demonstrated that cowpea-maize intercropping could deplete HCl-extracted P_i_ fractions relative to monocropping in semi-arid soils under convention NPK fertilization, which probably resulted from a combination of microbial and rhizosphere processes. However, the biochemical driving mechanisms involved in maize-soybean intercropping remain unclear, thus deserving further investigation.

The Huang-Huai-Hai Plain is one of the main crop producing area in China. The soil in this area is generally rich in carbonates and clay minerals, which strongly react with P compounds through sorption and precipitation, resulting in low P use efficiency of applied phosphate fertilizer (Liu et al., 2019). Therefore, the objectives of this study were to: (1) Identify the transformation of P fractions; (2) investigate chemical and biological factors driving the transformation of specific P fractions, and (3) explore the bacterial community composition under maize-soybean intercropping.

## Materials and methods

### Experimental design and sampling

A pot experiment was conducted in a greenhouse where one maize (*Zea mays* L.) and one soybean [*Glycine max* (L.) Merr.] were planted in rhizoboxes. Three treatments of root interactions were set up with three replicates: maize-soybean intercropping with no barrier (NB); with nylon net isolation (mesh barrier, MB, pore size = 40 μm) to prevent root interactions between different crops, but allow root exudate exchange; and plastic plate isolation (solid barrier, SB) to simulate monocropping. The soil used for the pot experiment is classified as Inceptisols according to the USDA Soil Taxonomy (USDA, 1999). The rhizoboxes (28 × 14 × 18 cm) were filled with 6 kg of air-dried soil and moistened to 50% of the maximum water holding capacity. The crops were sown directly into each rhizobox and no extra nutrients were applied during the whole growth period.

Plants were harvested after growing for 3 months. The shoots were oven-dried at 105°C for 0.5 h and dried to constant weight at 60°C, then weighed for biomass determination and finely ground for total P measurement by inductively coupled plasma optical emission spectroscopy (ICP-OES, Agilent 5110) after nitric acid-perchloric acid digestion (Smith and Joynson, 1974). The residual plants with roots were manually removed from the soil, and then shaken gently to remove loosely adhering soil from the roots. The rhizosphere soil was collected by vigorously shaking and gently brushing the root system (Yang et al., 2010). Soil from an unplanted treatment was also sampled as the bulk soil (BS). A portion of each soil sample was air-dried and sieved (<2 mm) for the measurements of soil properties and sequential P fractionation. Sub-samples were stored at 4°C for enzyme assays, and –80°C for DNA sequencing.

### Chemical analysis

Soil pH was measured in deionized water (1:2.5 w/v; Lu, 1999). Total carbon (C) and total N (TN) were determined by element analyzer (Vario Max). Prior to organic C (OC) measurement, soil samples were pre-treated with hydrochloric acid to remove inorganic C (IC). Soil IC was calculated as the difference between TC and OC. Total P was determined by H_2_SO_4_-HClO_4_ digestion, P_o_ was determined by ignition, both analyzed colorimetrically using the molybdate blue method (Murphy and Riley, 1962). The alkaline phosphomonoesterase (ALP) and phosphodiesterase (PDE) activities were measured by *p*-nitrophenyl phosphate as substrate with the buffer pH 11.0, and bis-*p*-nitrophenyl phosphate as substrate with the buffer pH 8.0, respectively (Tabatabai, 1994).

Soil P fractions were extracted by the fractionation scheme recommended by Jiang and Gu (1989). Soil samples was sequentially extracted by (1) 0.25 mol L^–1^ NaHCO_3_ at pH 7.5 [calcium (Ca)_2_-P]; (2) 0.5 mol L^–1^ NH_4_Ac at pH 4.2 (Ca_8_-P); (3) 0.5 mol L^–1^ NH_4_F [aluminum (Al)-P]; (4) 0.1 mol L^–1^ NaOH-Na_2_CO_3_ at pH 8.2 [iron (Fe)-P]; (5) 0.3 mol L^–1^ citrate solution and Na_2_S_2_O_4_ kept at 40°C [occluded (O)-P]; (6) 0.25 mol L^–1^ H_2_SO_4_ (Ca_10_-P). Ca_2_-P extracted by bicarbonate is categorized as labile. The identifications of each fraction (“Ca_2_-P,” “Al-P,” etc.) are those indicated in the fractionation protocol; we acknowledge that these fractions were operationally defined, and may likely contain more than one P compound. The concentration of molybdate-reactive P, considered here to be P_i_, in each extract was determined by colorimetric analysis within 24 h (Murphy and Riley, 1962), and the total P concentration in each extract was analyzed by ICP-OES. Molybdate-unreactive P concentrations were calculated as the difference between total P and P_i_, and for this study is considered to be P_o_.

### Illumina MiSeq sequencing of bacteria

Soil DNA was extracted using the E.Z.N.A.^®^ Soil DNA Kit (Omega Bio-tek, Norcross, GA, U.S.) according to manufacturer’s protocol, and quantified with a NanoDrop 2000 spectrophotometer. The primers 338F (5′-ACTCCTACGGGAGGCAGCAG-3′) and 806R (5′-GGACTACHVGGGTWTCTAAT-3′) were used to amplify the V3-V4 region of the 16S rRNA genes by an ABI GeneAmp^®^ 9700 PCR thermocycler. The amplification was performed in triplicates as follows: 3 min initial denaturation at 95°C, followed by 27 cycles of 95°C for 30 s, 55°C for 30 s, and 72°C for 45 s, with a final 10 min extension at 72°C, and stored at 4°C. The PCR mixtures contain 4 μL of 5 × TransStart FastPfu buffer, 2 μL 2.5 mM dNTPs, 0.8 μL forward primer (5 μM), 0.8 μL reverse primer (5 μM), 0.4 μL TransStart FastPfu DNA Polymerase, 10 ng template DNA, and finally ddH_2_O up to 20 μL. The PCR product was extracted from a 2% agarose gel, purified using the AxyPrep DNA Gel Extraction Kit (Axygen Biosciences, Union City, CA, USA), and quantified using Quantus Fluorometer (Promega, USA). The purified amplicons were pooled in equimolar and paired-end sequenced on an Illumina MiSeq PE300 platform (Illumina, San Diego, USA) according to the standard protocols by Majorbio Bio-Pharm Technology Co., Ltd. (Shanghai, China).

Raw sequence reads were processed using the Quantitative Insights into Microbial Ecology (QIIME2) pipeline (Caporaso et al., 2010). The adaptor sequences, barcodes and low-quality bases at the end of each read were removed. The raw paired-end reads were quality-filtered by fastp (version 0.19.6) and merged by FLASH (version 1.2.7; Magoc and Salzberg, 2011), with the following criteria: (i) The maximum mismatch ratio of overlap region is 0.2. Reads that could not be assembled were discarded; (ii) only overlapping sequences longer than 10 bp were assembled according to their overlapped sequence. The operational taxonomic units (OTUs) were clustered with 97% similarity cutoff using UPARSE version 7.1 (Edgar, 2013). Totally, there were 941,784 optimized sequences (392,730,025 bp) in 21 samples. A representative sequence of each OTU was analyzed by RDP Classifier version 2.2 (Wang, 2007) against the 16S rRNA gene database (Silva v138) using confidence threshold of 0.7. To avoid potential bias caused by differences in sequences depth, the number of sequences in each sample were rarefied to 28,570 sequences, which still yielded an average Good’s coverage of 97.61%, respectively. The raw reads were deposited into the NCBI Sequence Read Archive (SRA) database (Accession Number: PRJNA782812).

### Statistical analysis

The P fractionation data were centered log-ratio transformed before the statistical analysis (Liu et al., 2015). For the data of soil properties, P fractionation and plant biomass as well as P uptake, one-way analysis of variance (ANOVA) was conducted using SPSS 25 software (SPSS, Inc.), followed by a least significant difference (LSD) test with α = 0.05. Bioinformatic analysis of the soil was conducted by Majorbio Cloud^1^. Mothur v1.30.2 to identify them, using “classify.otu.” Rarefaction curves (Supplementary Figure 2), Chao richness, Shannon and Simpson indices were calculated at the 0.03 cut off level to assess the alpha diversity (Cole et al., 2009). The “Stats” package in R and “Scipy” in python were used to test the differences in the relative abundance of OTUs or genera between treatments using the Benjamini-Hochberg *P*-value correction. And the differences were analyzed with a one-way ANOVA followed by Tukey’s honestly significant difference (HSD) test. The data means were considered significantly different at *P* < 0.05. “Pheatmap” package in R was conducted to calculate the correlation coefficient between environmental factors and the selected species. Both hierarchical cluster method of environmental factors and species were average. The co-occurrence networks were constructed to explore the internal community relationships across the samples (Barberan et al., 2012). The network was constructed by Spearman correlation among genera with the absolute value of that correlation coefficient ≥ 0.5 and *P* < 0.05, which was analyzed by Networkx (python package Hagberg et al., 2008). We reconstructed a Maximum likelihood (ML) phylogeny based on the aligned OTU sequences of Silva collections using the FastTree (version 2.1.3)^2^. The ML phylogeny was visualized using the R (version 3.3.1).

## Results

### Plant biomass and P uptake

Maize-soybean intercropping generally enhanced aboveground biomass and total P uptake of crops compared to monocropping (Table 1). For maize, plant biomass and P uptake were highest in the NB treatment followed by the MB treatment, both of which were higher than the SB treatment. For soybean, plant biomass and P uptake were highest in the MB treatment and lowest in the NB treatment. For the sum of two crops, total biomass and P uptake were significantly higher for the MB and NB treatments compared to the SB treatment.

**TABLE 1:** Aboveground biomass and total phosphorus (P) uptake of maize and soybean under the different separation treatments*^a^* (means ± standard errors, *n* = 3).

	Aboveground biomass (g pot^–1^)			Total P uptake (mg pot^–1^)		
	Maize	Soybean	Sum*^b^*	Maize	Soybean	Sum*^b^*
SB	16.5 ± 0.7 c	10.1 ± 0.3 b	26.6 ± 0.5 b	20.3 ± 1.0 c	26.4 ± 1.3 b	46.7 ± 1.6 b
MB	25.6 ± 0.8 b	13.4 ± 1.0 a	39.0 ± 1.8 a	34.6 ± 2.4 b	41.2 ± 3.5 a	75.8 ± 5.9 a
NB	35.0 ± 1.3 a	7.41 ± 0.8 c	42.4 ± 0.9 a	46.9 ± 1.8 a	15.0 ± 2.4 c	62.0 ± 4.2 a

^a^SB, solid barrier; MB, mesh barrier; NB, no barrier.

^b^Sum, sum of maize and soybean plant material.

### Soil properties

For the BS, pH was 8.5 and TN, TC, OC, and IC concentrations were 0.07, 1.74, 0.59, and 1.14%, respectively (Table 2). Total P was 773.4 mg kg^–1^ in the BS where a small concentration of 83.5 mg kg^–1^ was P_o_. The ALP and PDE activities in the BS were 176.3 and 14.6 μg g^–1^h^–1^ (Table 2). Maize and soybean monocropping and intercropping significantly affected soil chemical and biological properties. Compared to the BS, maize monocropping (SB) significantly reduced rhizosphere soil pH but increased P_o_ contents and PDE activities (Table 2). Similar changes were also observed for soybean monocropping (SB) relative to the BS (Table 2). Compared to the SB, the NB treatment increased TN, TC, OC, P_o_, as well as ALP and PDE activities, but decreased IC contents in the rhizosphere soils for both maize and soybean (Table 2). For both crops, higher ALP and PDE activities occurred in the MB treatment than the NB treatment (Table 2). A larger concentration of P_o_ in the MB treatment was shown compared to the NB treatments for soybean, while this change was insignificant for maize (Table 2).

**TABLE 2:** Chemical properties*^a^* of the bulk and rhizospheric soils of the maize and soybeans under the different separation treatments*^b^* (means ± standard errors, *n* = 3)*^c^*.

		pH	TN (%)	TC (%)	OC (%)	IC (%)	TP (mg⋅kg^–1^)	P_o_ (mg⋅kg^–1^)	ALP (μg⋅g^–1^⋅h^–1^)	PDE (μg⋅g^–1^⋅h^–1^)
BS		8.50 ± 0.06 a A	0.07 ± 0.11 b B	1.74 ± 0.19 c B	0.59 ± 0.02 c B	1.14 ± 0.04 a A	773.4 ± 5.3 a A	83.5 ± 6.7 c D	176.3 ± 9.32 c C	14.6 ± 0.41 c B
Maize	SB	8.06 ± 0.01 b	0.07 ± 0.00 b	1.80 ± 0.03 bc	0.65 ± 0.05 bc	1.15 ± 0.17 a	768.6 ± 3.3 a	122.9 ± 0.9 b	159.6 ± 7.58 c	24.9 ± 1.2 b
	MB	8.12 ± 0.03 b	0.08 ± 0.01 b	1.84 ± 0.02 ab	0.79 ± 0.08 b	1.06 ± 0.57 a	784.5 ± 21.3 a	128.6 ± 2.8 b	278.8 ± 11.44 b	34.2 ± 4.79 a
	NB	8.19 ± 0.03 b	0.10 ± 0.00 a	1.91 ± 0.05 a	1.14 ± 0.01 a	0.77 ± 0.01 b	777.7 ± 9.7 a	158.9 ± 5.4 a	357.4 ± 5.35 a	41.6 ± 2.14 a
Soybean	SB	8.23 ± 0.04 B	0.06 ± 0.00 B	1.75 ± 0.02 B	0.59 ± 0.01 B	1.16 ± 0.01 A	747.1 ± 4.2 B	99.4 ± 1.2 C	192.7 ± 3.39 C	20.7 ± 1.01 B
	MB	8.28 ± 0.05 B	0.07 ± 0.01 B	1.77 ± 0.01 B	0.68 ± 0.07 B	1.09 ± 0.06 A	762.8 ± 4.2 A	116.3 ± 2.9 B	245.1 ± 10.60 B	32.3 ± 3.74 A
	NB	8.50± 0.06 AB	0.11 ± 0.00 A	1.92 ± 0.02 A	1.12 ± 0.02 A	0.80 ± 0.01 B	764.8 ± 3.3 A	134.7 ± 1.3 A	333.1 ± 4.36 A	37.9 ± 0.59 A

^a^TN, total nitrogen; TC, total carbon; OC, organic carbon; IC, inorganic carbon; TP, total P; P_o_, organic P; ALP, alkaline phosphatase; PDE, phosphodiesterase.

^b^ BS, bulk soil; SB, solid barrier; MB, mesh barrier; NB, no barrier.

^c^Values in each column followed by the same lowercase letters or the same uppercase letters are not significantly different (*P* < 0.05). The lowercase letters represented the difference among the treatments of maize rhizosphere soil and bulk soil, while the uppercase letters represented the difference among the treatments of soybean rhizosphere soil and bulk soil.

### Soil P transformation

Sequential fractionation recovered 71.7–75.3% of P_i_ (Table 3). The majority of soil P_i_ was extracted by H_2_SO_4_ (Ca_10_-P_i_), followed by NH_4_Ac (Ca_8_-P_i_). Phosphorus transformation was induced in the rhizosphere of both crops by monocropping and intercropping. In the monocropping (SB) treatment, Al-P_i_ and O-P_i_ significantly decreased in the maize rhizosphere soil, and Ca_8_-P_i_ and Fe-P_i_ significantly decreased in the soybean rhizosphere soil, respectively, compared to the BS; there were no significant changes in the other P_i_ fractions (Table 3). Under the intercropping treatment with no barrier, there were significant decreases of Ca_2_-P_i_, Al-P_i_, Fe-P_i_, but a significant increase of Ca_8_-P_i_ in the rhizosphere soils of the two crops compared to the SB (Table 3). Similarly, this effect was observed in the MB treatment with higher Ca_8_-P_i_, and O-P_i_ for maize and higher Ca_8_-P_i_ and lower O-P_i_ for soybean than the SB soil.

**TABLE 3:** Phosphorus (P) fractions in the bulk and rhizospheric soils of the maize and soybean under the different separation treatments*^a^* (mean ± standard errors, *n* = 3)*^b^*.

		NaHCO_3_-P	NH_4_Ac-P	NH_4_F-P	NaOH-Na_2_CO_3_-P	Citrate-dithionite-P	H_2_SO_4_-P	Recovery
		———————————————————mg kg^–1^———————————————————						%
**Inorganic phosphorus**								
BS		2.6 ± 0.2 a A*^b^*	59.6 ± 1.0 c C	27.7 ± 0.3 a A	27.6 ± 0.2 a A	9.1 ± 0.2 a A	371.2 ± 0 a A	72.2
Maize	SB	2.6 ± 0.2 a	58.7 ± 1.5 c	25.3 ± 0.3 b	26.3 ± 0.7 a	2.3 ± 0 d	368.2 ± 6.4 a	74.9
	MB	2.2 ± 0.2 a	66.4 ± 1.3 b	26.6 ± 0.6 a	24.4 ± 0.7 a	3.5 ± 1.0 c	360.8 ± 5.3 a	73.9
	NB	1.3 ± 0.1 b	74.3 ± 2.7 a	20.1 ± 0.4 c	17.5 ± 0.7 b	5.1 ± 0.4 b	344.1 ± 4.7 a	74.8
Soybean	SB	2.2 ± 0 AB	52.9 ± 0.2 D	26.0 ± 0.2 A	24.4 ± 0.5 B	8.9 ± 0.2 A	373.2 ± 2.9 A	75.3
	MB	2.0 ± 0.1 B	65.0 ± 1.1 B	25.7 ± 0.3 A	23.2 ± 0.8 B	8.0 ± 0.1 B	378.6 ± 2.5 A	77.7
	NB	1.3 ± 0 C	69.6 ± 4.8 A	19.4 ± 0.1 B	17.7 ± 0.8 C	6.4 ± 0.1 C	337.3 ± 4.2 A	71.7
**Organic phosphorus**								
BS		6.4 ± 0.4 a A	ND*^c^*	12.9 ± 1.1 b A	50.3 ± 1.6 c C	4.5 ± 0.3 b C	ND	89.7
Maize	SB	7.3 ± 0.8 a	ND	18.4 ± 0.7 a	59.7 ± 0.5 b	10.6 ± 0.3 a	ND	78.3
	MB	5.1 ± 0.9 a	ND	14.4 ± 0.7 b	61.0 ± 1.9 b	8.9 ± 1.2 a	ND	69.6
	NB	5.9 ± 0.4 a	ND	12.7 ± 0.9 b	84.5 ± 0.6 a	8.1 ± 0.6 a	ND	70.1
Soybean	SB	4.8 ± 0.1 A	ND	15.8 ± 0.1 A	52.5 ± 0.9 C	5.1 ± 0.5 BC	ND	78.7
	MB	5.1 ± 0.3 A	ND	12.7 ± 1.5 A	60.3 ± 2.2 B	6.1 ± 0.5 B	ND	72.5
	NB	5.0 ± 0.7 A	ND	9.0 ± 0.6 B	83.5 ± 0.7 A	7.3 ± 0 A	ND	77.9

^a^BS, bulk soil; SB, solid barrier; MB, mesh barrier; NB, no barrier.

^b^Values in each column followed by the same lowercase letters or the same uppercase letters are not significantly different (*P* < 0.05). The lowercase letters represented the difference among the treatments of maize rhizosphere soil and bulk soil, while the uppercase letters represented the difference among the treatments of soybean rhizosphere soil and bulk soil.

^c^ND, not detected.

Fractionation recovered 69.6–89.7% of P_o_, most of which was Fe-P_o_ (Table 2). For the SB treatment, there were significant increases in the Al-P_o_, Fe-P_o_ and O-P_o_ contents, but no significant changes in the Ca_2_-P_o_ content in the maize rhizosphere relative to the BS treatment (Table 3). In contrast, there were no significant differences in the detected P_o_ fractions in the soybean rhizosphere soil under the SB treatment compared to the BS (Table 3). Compared to the SB, there were significant decreases in Al-P_o_ and increases in the Fe-P_o_ fractions in the rhizosphere soils of both crops under the NB treatment, with no significant changes in the Ca_2_-P_o_ content (Table 3). Consistently, the decreased Al-P_o_ for maize and increased Fe-P_o_ for soybean occurred under the MB treatment compared to the SB (Table 3).

### Soil bacterial community composition

For both maize and soybean, soil bacterial richness (Chao) was significantly decreased in the intercropping (NB vs. SB treatment) rather than the monocropping system (SB vs. BS treatment; Supplementary Table 1). In contrast, the Shannon index was not affected by intercropping or monocropping (except for maize; Supplementary Table 1). Moreover, Actinobacteriota, Proteobacteria, Chloroflexi, and Acidobacteriota were the main phyla for all soil treatments of the two crops (Supplementary Figure 1).

**FIGURE 1:**
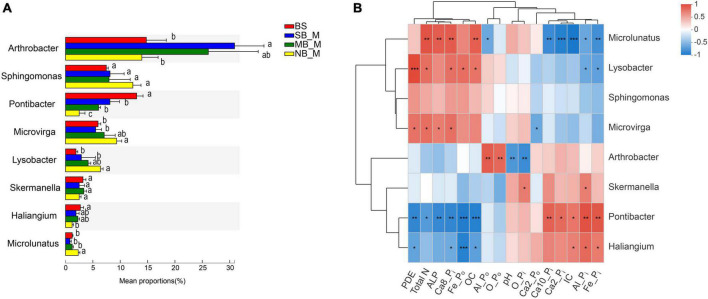
Abundances of the selected bacteria in the bulk and maize rhizosphere soils at the genus level under different separation treatments **(A)** and their correlation with selected P fractions and soil properties **(B)**. BS, bulk soil; SB, solid barrier; MB, mesh barrier; NB, no barrier. Values marked by the same lowercase letters in Figure 1A are not significantly different among different separation treatments (*P* < 0.05). *P < 0.05, **P < 0.01, ***P < 0.001.

**FIGURE 2:**
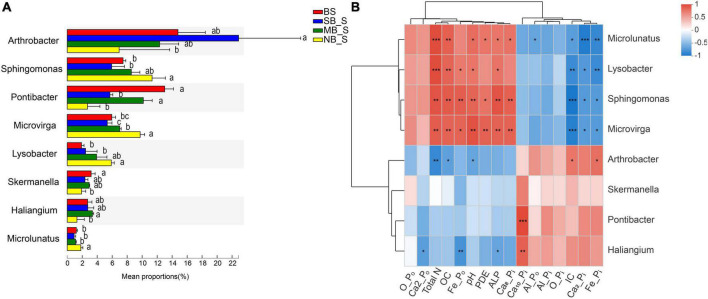
Abundances of the selected bacteria in the bulk and soybean rhizosphere soils at the genus level under different separation treatments **(A)** and their correlation with selected P fractions and soil properties **(B)**. BS, bulk soil; SB, solid barrier; MB, mesh barrier; NB, no barrier. Values marked by the same lowercase letters in Figure 2A are not significantly different among different separation treatments (*P* < 0.05). *P < 0.05, **P < 0.01, ***P < 0.001.

**FIGURE 3:**
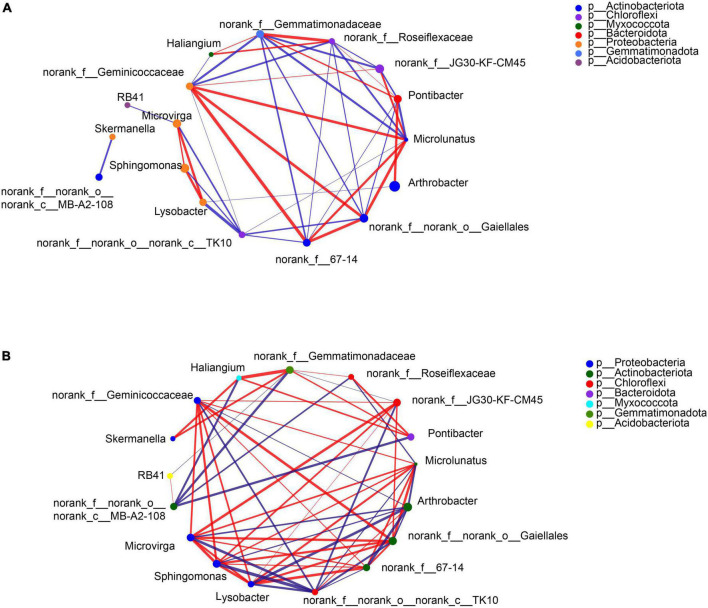
Correlation network of the selected bacteria at the genus level in the maize **(A)** and soybean **(B)** rhizosphere soils under intercropping.

Specific bacteria showed significant differences in relative abundances at the genus level in the rhizosphere soils of the two crops under different separation treatments. Compare to the BS, the SB treatment significantly reduced the abundance of *Pontibacter* in the rhizosphere of the two crops, and increased the abundance of *Arthrobacter* in the maize rhizosphere (Figures 1A, 2A). In contrast, there was a significant decrease in the abundance of *Arthrobacter* but significant increases in the abundances of *Microvirga, Lysobacter* and *Microlunatus* in the rhizosphere soils of the two crops under the NB treatment relative to the SB treatment (Figures 1A, 2A). Similarly, the abundance of *Sphingomonas* in the soybean rhizosphere soil significantly increased under the NB treatment compared to the SB treatment (Figure 2A). For the MB treatment, the abundance of each selected bacterial genus (*Arthrobacter*, *Sphingomonas*, *Microvirga*, *Lysobacter*, *Microlunatus, Skemanella, Haliangium*) except *Pontibacter*, showed a transition trend compared to the NB and SB treatments (Figures 1A, 2A). Additionally, certain non-ranked bacteria phyla were also found under maize-soybean intercropping, which had close genetic relationships with the aforementioned bacteria related to P cycling (Supplementary Figure 3). For all bacteria involved in P cycling, including the non-ranked bacteria, a tighter engagement in the network of these bacteria was observed in the rhizosphere of soybean than maize (Figure 3).

The correlation analyses of P fractions, soil biochemical properties and the abundances of selected bacteria genera were conducted for the rhizosphere soils of the two crops. Abundance of *Pontibacter* was positively correlated with concentrations of Al-P_i_, Ca_2_-P_i_, Fe-P_i_, Ca_10_-P_i_ and IC, but negatively correlated with concentrations of OC, Ca_8_-P_i_, TN, ALP, and Fe-P_o_ in the maize rhizosphere soils (Figure 1B). In contrast, the abundance of *Pontibacter* was positively correlated only with the Ca_10_-P_i_ concentration in the soybean rhizosphere soils (Figure 2B). Furthermore, the abundance of *Arthrobacter* exhibited positive correlations with Al-P_o_ and O-P_o_ concentrations, but negative correlations with the O-P_i_ concentration and pH in the maize rhizosphere soils (Figure 1B), and positive correlations with IC and Fe-P_i_ concentrations, but negative correlations with TN and OC concentrations and pH in the soybean rhizosphere soils (Figure 2B). Moreover, *Microvirga* and *Lysobacter* both only had positive correlations with TN, PDE and Ca_8_-P_i_ concentrations in the maize rhizosphere soil (Figure 1B), but were positively correlated with TN, OC, Fe-P_o_, concentration, pH, and ALP activity but were negatively correlated with IC, Ca_2_-P_i_, Fe-P_i_ concentrations in the soybean rhizosphere soil (Figure 2B). For both crops, the abundance of *Microlunatus* positively correlated with TN, OC, ALP and Ca_8_-P_i_ concentrations, but negatively correlated with Al-P_o_, IC, Ca_2_-P_i_, Fe-P_i_ concentrations (Figures 1B, 2B). In addition, the abundance of *Sphingornonas* showed no significant correlations with any soil P fractions and biochemical properties in the maize rhizosphere soils (Figure 1B). However, it was positively correlated with TN, pH, OC, Ca_8_-P_i_ and Fe-P_o_ concentrations as well as ALP and PDE activities, and was negatively correlated with IC, Ca_2_-P_i_ and Fe-P_i_ concentrations in the soybean rhizosphere soils (Figure 2B).

## Discussion

### Intercropping facilitated transformation of P fractions

Maize-soybean intercropping significantly promoted crop growth and P uptake, as indicated by the increase of the total above-ground biomass and P uptake of the two crops (maize and soybean combined, Table 1). These results agreed with previous reports showing that maize-legume intercropping benefited crop production and P accumulation (Betencourt et al., 2012; Liao et al., 2020). The lowest biomass and P uptake of soybean but highest of maize under the NB treatment relative to the SB treatment (Table 1) resulted from the competition for soil nutrients between maize and soybean, and maize could absorb more nutrients than soybeans (Zhang et al., 2015). Generally, most cereal/legume polycultures are asymmetric and thus lead to imbalanced nutrient absorption by crops (Zhang et al., 2016).

Besides enhancing crop P uptake, maize-soybean intercropping induced significant transformation of P fractions in this calcareous soil. This was indicated by the significant decrease in concentrations of Ca_2_-P_i_, Al-P_i_/P_o_ and Fe-P_i_, but significant increases in Ca_8_-P_i_ and Fe-P_o_ concentrations in the rhizospheres of maize and soybeans under intercropping (NB) relative to monocropping (SB, Table 3). However, compared to the bulk soil, only concentrations of Al-P_i_ and O-P_i_ in the maize rhizosphere soil and Ca_8_-P_i_ and Fe-P_i_ in the soybean rhizosphere soil significantly decreased, while Al-P_o_, Fe-P_o_ and O-P_o_ concentrations significantly increased in the maize rhizosphere soil under monocropping (SB, Table 3). These results indicate that maize monoculture improved the recovery of P from Al-P_i_ and O-P_i_ fractions, while soybean monoculture could mobilize P from Ca_8_-P_i_ and Fe-P_i_ pools. In contrast, maize-soybean intercropping could further utilize the Ca_2_-P_i_ fraction, in addition to Al-P_i_ and Fe-P_i_ fractions. Generally, the Ca_2_-P_i_ fraction, as the most bioavailable P fraction to crops, could be supplied from the diffusion of labile P from the bulk soil P pools to the rhizosphere (Hinsinger, 2001). The depletion of rhizospheric Ca_2_-P_i_ fraction under maize-soybean intercropping rather than maize or soybean monoculture highlighted the stronger recovery capacity of soil legacy P under intercropping relative to monoculture. Moreover, the significant decrease of Al-P_i_ and Fe-P_i_ fractions in the rhizosphere of both crops under the NB treatment relative to the SB treatment further highlights that maize-soybean intercropping facilitates the recovery of P sorbed to Al/Fe oxides. This probably resulted from the rhizosphere effects of maize-soybeans, which could mobilize micronutrients such as Fe in calcareous soils (Dragicevic et al., 2015). Although reports on the influence of maize-soybean intercropping on soil P transformations are scarce in the literature, one field study of 4-year maize-legume (i.e., faba bean) intercropping without P fertilization suggests intercropping could significantly deplete NaOH-extracted P_i_ fraction, generally assigned as P_i_ associated with Fe/Al oxides (Liu et al., 2013), in calcareous soil compared to monocropping (Liao et al., 2020). Additionally, maize-soybean intercropping also resulted in the rhizospheric accumulation of Fe-P_o_ and decrease of Al-P_o_ fractions for the two crops (Table 3). These results indicated maize-soybean intercropping facilitated the transformation of P_i_ fractions (Ca_2_-P_i_, Al-P_i_, and Fe-P_i_) into Fe-P_o_ fractions in the calcareous soil, which agreed with the increased rhizospheric P_o_ contents for the two crops under the NB treatment (Table 2).

### Chemical and biochemical factors affecting P transformation under maize-soybean intercropping

Maize-soybean intercropping could increase soil P availability through multiple mechanisms including root exudates and proton-induced dissolution of P-containing minerals, increased phosphatase activity, and functional microbe-mediated P_i_ mobilization and P_o_ mineralization (Hinsinger, 2001; Betencourt et al., 2012; Amadou et al., 2021; Park et al., 2022). In this study, rhizospheric OC content significantly increased for both crops under the NB treatment relative to the SB treatment (Table 2), which reflected the accumulation of rhizosphere OC under the NB treatment. Rhizosphere OC deposition has multiple sources including root exudates, microbial turnover and litter decomposition (Hinsinger et al., 2009), and root exudates were generally considered as the major driving force to induce the dissolution of solid phase associated P_i_ fractions including Ca_2_-P_i_, Al-P_i_ and Fe-P_i_ (Table 3; Hinsinger, 2001). Generally, all three aforementioned P_i_ fractions are sensitive to low molecular-weight organic acids, which, as one of the major components in root exudates, could dissolve brushite, Al and Fe oxides through ligand-induced complexation and dissolution (Qin et al., 2013, 2018). Furthermore, phytosiderophores and phenolic acid were widely reported to be present in the root exudates of maize and legume, respectively (Bekkara et al., 1998; Bernards et al., 2002; Wang et al., 2014), which could destroy Fe oxides minerals and mobilized their associated P_i_ (Hunt et al., 2007; Reichard et al., 2007). This probably also accounted for the decrease of Fe-P_i_ fraction in the rhizosphere soils of both crops (Table 3). Moreover, the enhanced rhizospheric TN contents caused by soybean-mediated N fixation processes in the maize-soybean intercropping would also be beneficial to soil P mobilization (Tables 2, 3). There were several potential driving factors derived from the soybeans, including the production of root exudates and phosphatases and enhanced proton release during N_2_-fixation (Betencourt et al., 2012). In this study, rhizospheric acidification was excluded as the major driving factor for the observed transformation of P fractions under the maize-soybean intercropping, given the lack of significant changes in the rhizospheric pH for both crops among the three separation treatments (Table 2). The high pH buffer capacity of the investigated calcareous soil probably resulted in the resistance of rhizospheric acidification in this soil under the intercropping (Andersson et al., 2015). Therefore, the accumulation of rhizosphere OC deposit and phosphatases probably contributed to the recovery of soil P_i_ fractions, which agreed with the enhanced OC contents and phosphatase activities under the maize-soybean intercropping (Table 2).

For the cycling of P_o_ pools, enzymes serve as one of the major driving factors to mineralize P_o_ into the available P_i_ pool for crop uptake (Richardson et al., 2009; Park et al., 2022; Yu et al., 2022). Generally, the ALP in soils is considered as microbial origin, while the PDE could be from plant roots and microbes (Amadou et al., 2021). Compared to the SB treatment, the enhanced ALP and PDE activities in the rhizosphere soils of both crops under the NB treatment (Table 2) implies increased mineralization of soil P_o_ fractions. However, the net accumulation of P_o_ contents under the NB treatment indicated that mineralization rates of soil P_o_ fractions were less than the accumulation rates of P_o_ fractions under the maize-soybean intercropping. The depletion of the Al-P_o_ fraction in the rhizosphere soils of both crops under the NB treatment (Table 3) was less likely related to enhanced enzyme-mediated P_o_ mineralization; instead, it probably resulted from ligand-induced dissolution of Al oxides by root exudates considering the enhanced rhizospheric OC content of the two crops under the NB treatment relative to the SB treatment (Table 2). Additionally, the enrichment of the Fe-P_o_ fraction in the rhizosphere soils of both crops under maize-soybean intercropping probably resulted from the rhizospheric transformation of crystalline Fe oxides to amorphous phase, which have relatively high surface area and in turn enhance P_o_ immobilization and accumulation (Liu et al., 2015). A previous long-term field study also found increased P concentration in the Fe-P_o_ fractions of soil under long-term maize monocropping (Liu et al., 2017).

### Bacteria affecting rhizospheric P transformation under maize-soybean intercropping

Maize-soybean intercropping selectively enriched specific bacterial phyla in the rhizosphere (Figures 1A, 2A). The enrichment of *Microvirga, Lysobacter, and Microlunatus* in the rhizosphere of both crops under the NB treatment suggests that maize and soybean could harbor certain bacterial genera in their rhizosphere soils under intercropping. *Microvirga* (Rhizobiales), as an N-fixing bacteria (Pathan et al., 2020; Zhang et al., 2021), could contribute to increased soil N concentrations and subsequent plant growth, which probably indirectly affected the mobilization of soil P as a result of enhanced release of root exudates (Betencourt et al., 2012). This agreed with the positive correlation of the abundance of *Microvirga* with the TN content in the rhizosphere soils of both crops (Figures 1B, 2B). Furthermore, the abundance of *Microvirga* was negatively correlated to the Ca_2_-P_o_ fraction but positively related to PDE and ALP activities. Legume roots, generally benefiting the colonization of N-fixing bacteria, also release high amounts of phosphatase (Betencourt et al., 2012), which probably contributed to the negative correlation between the *Microvirga* and Ca_2_-P_o_ fraction. Moreover, *Lysobacter* could release acid and alkaline phosphatases to degrade P_o_ (Chen et al., 2019). The enrichment of *Lysobacter* in the rhizosphere soils of the two crops under the NB treatment may have contributed to the enhanced PDE and ALP activity in the maize and soybean rhizospheres, respectively (Table 2). This implies that bacteria-mediated P_o_ cycling was enhanced in the rhizosphere of these crops, although the total P_o_ contents increased for both crops under the NB treatment (Table 2). In addition, *Microlunatus* facilitates the formation and accumulation of polyphosphate (Zhong et al., 2019). The enriched *Microlunatus* positively correlated with the Ca_8_-P_i_ fraction, but negatively correlated with the Ca_2_-P_i_, Al-P_i_ and Fe-P_i_ fractions in both maize and soybean rhizospheres (Figures 1B, 2B), suggesting that *Microlunatus* may be involved in transformation of Ca_2_-P_i_, Al-P_i_ and Fe-P_i_ fractions into the Ca_8_-P_i_ fraction through the formation of polyphosphate by *Microlunatus* and the subsequent hydrolysis of polyphosphate to replenish Ca_2_-P_i_, Al-P_i_ and Fe-P_i_ pools. A previous study also showed that the application of polyphosphate fertilizers increased the NaOH-extracted soil P fraction as a result of the relatively high solubility of polyphosphate (Shah and Chu, 2021). Since all the selected representative strains of these taxa related to P cycling were obtained from Illumina MiSeq sequencing analysis, future investigations on the isolation of representative strains of these taxa to test their functions are needed.

The presence of specific microorganisms also depended critically on the crop species among the root barrier treatments. There was a tighter interaction of bacteria, reported to be involved in P-cycling, in the rhizosphere of soybean than maize, which demonstrated the enhanced interactions of bacteria in the soybean rhizosphere (Figure 3). Furthermore, *Arthrobacter* was significantly enriched in maize monocropping (i.e., SB treatment) but was inhibited in the maize-soybean intercropping (i.e., NB treatment). The C–P lyase, detected in the *Arthrobacter* sp. (Kertesz et al., 1991), could contribute to the degradation of phosphonates by cleaving the C–P bond. This may have accounted for the higher P_o_ content in the maize-soybean intercropping relative to the maize monocropping. *Sphingomonas*, being able to utilize phytate (Sanangelantoni et al., 2018), accumulated in the rhizosphere of soybean but not for maize, which could account for the positive relationship of *Sphingomonas* with the Fe-P_o_ fraction enriched with orthophosphate monoester containing high abundance of phytate (Liu et al., 2013).

## Conclusion

This study provides new insights into the transformations of specific soil P fractions under maize-soybean intercropping and associated chemical and biological driving mechanisms. Maize-soybean intercropping facilitated the recovery of soil legacy P and induced the release of P from the Ca_2_-P_i_, Al-P_i_/P_o_ and Fe-P_i_ fractions, which probably resulted from synergetic effects of rhizosphere OC deposit, the enhanced enzymes (ALP and PDE) activities and the bacteria (*Microvirga*, *Lysobacter*, *Microlunatus*, and *Sphingomonas*) which exhibited correlation with soil P pools. The new finding that a tighter engagement in the network of the bacteria in the rhizosphere of soybean than that of maize suggests the potential application of soybean intercropping with cereal crops to recover soil legacy P resources. This facilitated the maintenance of soil fertility with low-carbon economics compared to conventional chemical fertilization, although future field scale studies are needed to confirm these results.

## Data availability statement

The data presented in this study are deposited in the Sequence Read Archive (SRA) of NCBI (https://www.ncbi.nlm.nih.gov/) (accession no. PRJNA782812).

## Author contributions

JL designed the study. CH and JL conducted the pot experiment. CH, YL, JL, and DY performed the data analyses. JL and YL wrote the manuscript with inputs from JY, BC-M, YC, and PS. All authors read and approved the manuscript.

## Footnotes

1
https://cloud.majorbio.com

2
http://www.microbesonline.org/fasttree/
