# Supplementary material for: Maize-soybean intercropping facilitates chemical and microbial transformations of phosphorus fractions in a calcareous soil
Source: Front Microbiol. 2022 Nov 16;13:1028969. doi: 10.3389/fmicb.2022.1028969 (PMC9709268; doi:10.3389/fmicb.2022.1028969)
Supplement: Supplementary file 1 [file Presentation_1.PDF]

Supplementary Material

Maize-Soybean Intercropping Facilitates Chemical and Microbial Transformations of Phosphorus Fractions in a Calcareous Soil

Jin Liu[1†*], Yang Li[1†], Chaoqun Han[1], Dongling Yang[1], Jianjun Yang[2], Barbara J. Cade-Menun[3], Yuanquan Chen[1], Peng Sui[1]

[1] College of Agronomy and Biotechnology, China Agricultural University, Beijing, 100094, China

[2] Institute of Environment and Sustainable Development in Agriculture, Chinese Academy of Agricultural Sciences, Beijing, 100081, China

[3] Agriculture & Agri-Food Canada, Swift Current RDC, Swift Current, SK, Canada

[†] These authors share first authorship

*** Correspondence:**
Jin Liu, China Agricultural University, College of Agronomy and Biotechnology, Beijing, 100094, China; jliu207@cau.edu.cn.

1. Supplementary Figures and Tables

1.1 Supplementary Figures

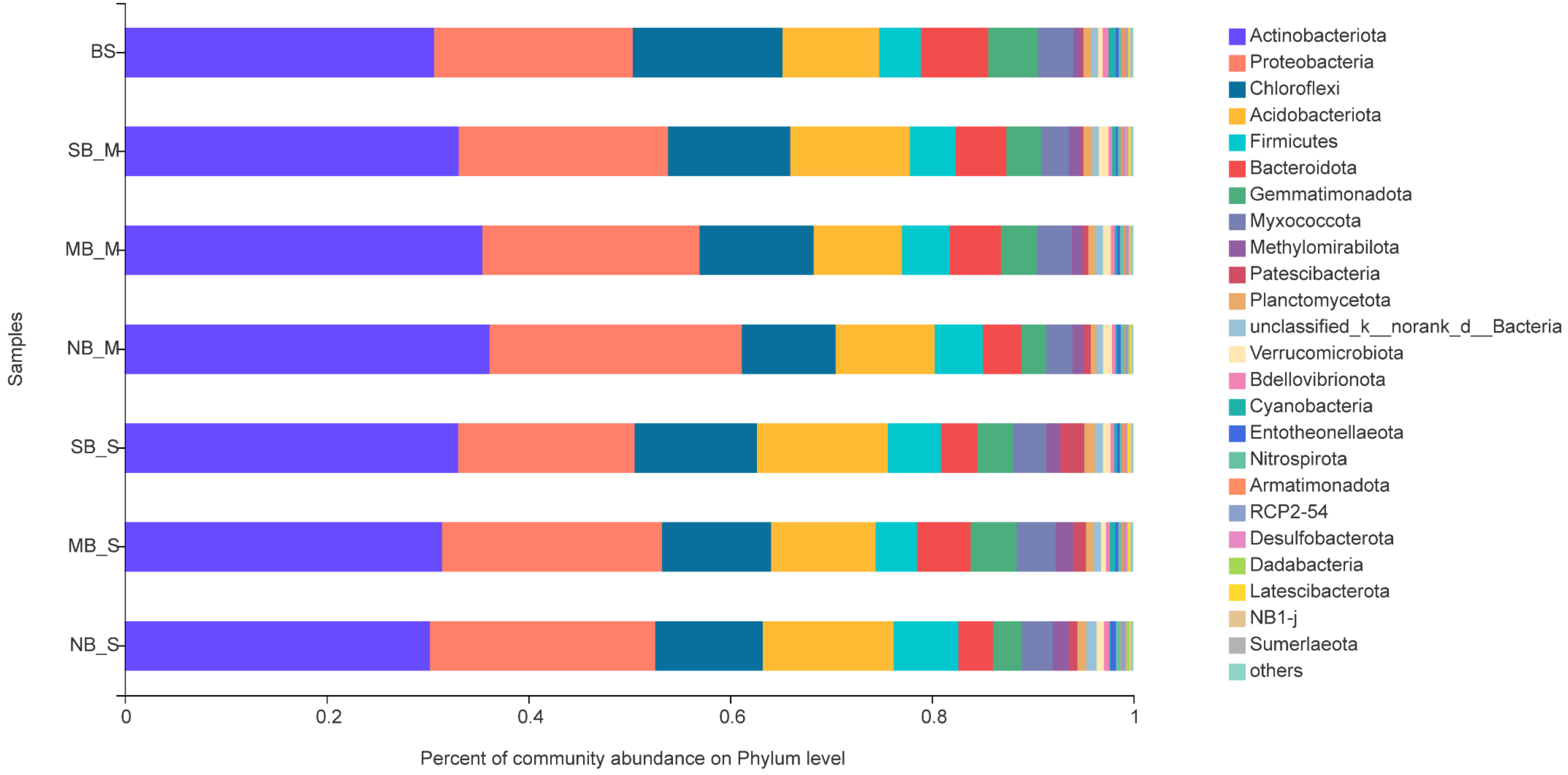

Figure S1 Relative abundance of bacteria in the maize (M) and soybean (S) soils under different separation treatments at the phylum level. BS: bulk soil; SB: solid barrier; MB: mesh barrier; NB: no barrier

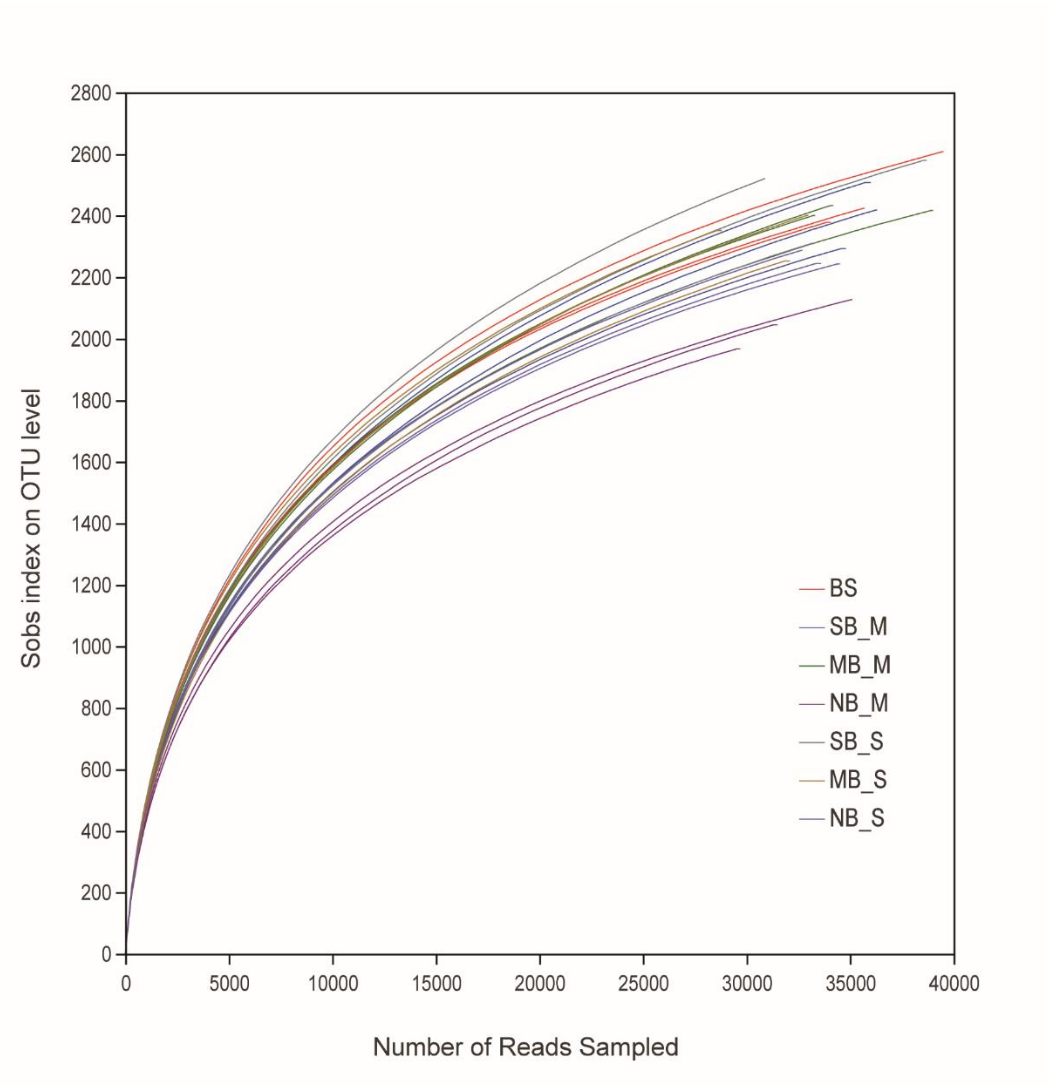

Figure S2 Rarefaction curve of bacterial at 0.03 cut-off level in the bulk and rhizospheric soils of the maize (M) and soybean (S) under the different separation treatments. BS: bulk soil; SB: solid barrier; MB: mesh barrier; NB: no barrier.

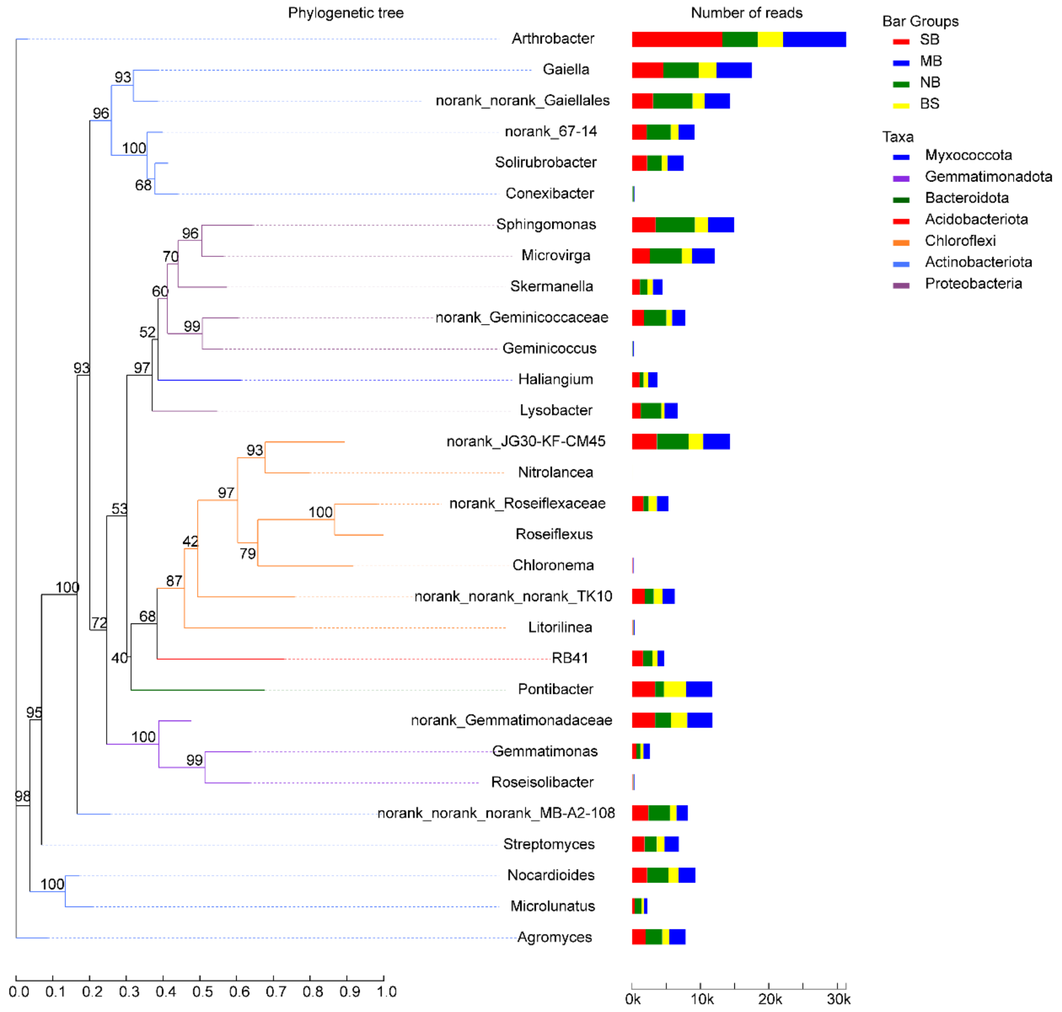

Figure S3 A phylogenetic representation for the bacteria which was significantly changed under intercropping. The colors on OTU stands for the phylum assignation. Abundances are transformed and displayed as heat maps. The OTUs assigned to generic level are shown by their taxonomic assignation.

1.2 Supplementary Tables

Table S1 Overview of 16S rRNA gene diversity and richness in the bulk and rhizospheric soils of the maize and soybean under the different separation treatments.

		Shannon	Simpson	Chao
	[a] BS	6.39 ± 0.03 a A	0.006 ± 0.001 b A	2937 ± 56.82 a AB
maize	SB	6.16 ± 0.05 bc	0.013 ± 0.002 a	3034 ± 82.67 a
	MB	6.25 ± 0.02 b	0.009 ± 0.001 ab	3009 ± 32.26 a
	NB	6.10 ± 0.04 c	0.009 ± 0.001 ab	2577 ± 21.97 b
soybean	SB	6.35 ± 0.08 A	0.007 ± 0.001 A	3137 ± 121.89 A
	MB	6.37 ± 0.05 A	0.005 ± 0.001 A	3028 ± 44.55 A
	NB	6.34 ± 0.01 A	0.005 ± 0.000 A	2762 ± 48.99 B

[a] BS: bulk soil; SB: solid barrier; MB: mesh barrier; NB: no barrier. [b] Values in each column followed by the same lowercase letters or the same uppercase letters are not significantly different ($P < 0.05$).
